# Detoxification Strategies for Zearalenone Using Microorganisms: A Review

**DOI:** 10.3390/microorganisms7070208

**Published:** 2019-07-21

**Authors:** Nan Wang, Weiwei Wu, Jiawen Pan, Miao Long

**Affiliations:** 1 Key Laboratory of Zoonosis of Liaoning Province, College of Animal Science & Veterinary Medicine, Shenyang Agricultural University, Shenyang 110866, China; 2 Institute of Animal Science, Xinjiang Academy of Animal Sciences, Urumqi 830000, China

**Keywords:** zearalenone (ZEA), reproductive toxicity, cytotoxicity, immunotoxicity, biological detoxification, probiotics, ZEA biotransformation

## Abstract

Zearalenone (ZEA) is a mycotoxin produced by *Fusarium* fungi that is commonly found in cereal crops. ZEA has an estrogen-like effect which affects the reproductive function of animals. It also damages the liver and kidneys and reduces immune function which leads to cytotoxicity and immunotoxicity. At present, the detoxification of mycotoxins is mainly accomplished using biological methods. Microbial-based methods involve zearalenone conversion or adsorption, but not all transformation products are nontoxic. In this paper, the non-pathogenic microorganisms which have been found to detoxify ZEA in recent years are summarized. Then, two mechanisms by which ZEA can be detoxified (adsorption and biotransformation) are discussed in more detail. The compounds produced by the subsequent degradation of ZEA and the heterogeneous expression of ZEA-degrading enzymes are also analyzed. The development trends in the use of probiotics as a ZEA detoxification strategy are also evaluated. The overall purpose of this paper is to provide a reliable reference strategy for the biological detoxification of ZEA.

## 1. Introduction

Zearalenone (ZEA) is a well-known F2 toxin that is produced by *Fusarium* fungi [1,2]. As it is one of the most widespread mycotoxins in the world [3,4,5], it not only affects food safety, but also accumulates in the food chain, causing serious harm to animals and even humans [6,7,8,9]. The detoxification of ZEA usually involves the use of some physical, chemical, or biological method to eliminate (or weaken) its toxicity [10,11,12]. However, the physical and chemical detoxification changes in the nutritional structure of the feed during the detoxification process, and the detoxification agents used may also cause secondary pollution to the environment. For example, adsorption using a mycotoxin adsorbent may yield species that are not particularly stable, and so the mycotoxin may be susceptible to desorption [13]. In addition, adsorbents containing aluminosilicates could, in principle, release components that are toxic (heavy metals or dioxins) [14].

In contrast, biological detoxification has high specificity, produces harmless products, and can even lead to complete detoxification under suitable conditions [15,16]. For example, it has been found that a strain of *Bacillus velezensis*, named A2, completely degraded ZEA (7.45 μg/mL) after three days of incubation at 37 °C in Luria-Bertani medium [17]. Furthermore, the microbial fermentation process employed can improve the nutritional structure of the feed and promote its transformation. The use of probiotics also improves the immune status of the livestock and poultry and therefore promotes their healthy growth [18,19,20,21,22]. As a result, ZEA detoxification using probiotics is a new technique with broad application prospects.

In this paper, the mechanisms by which ZEA causes damage to animals are revealed, and the microorganisms that can be used to detoxify ZEA are listed. The known biological mechanisms responsible for the detoxification of ZEA and the roles played by non-pathogenic microorganisms in ZEA detoxification are discussed in detail. Thus, the overall purpose of this paper is to clarify the mechanisms of ZEA biological detoxification, and then to provide a theoretical basis for the production and practical applications of ZEA microbial detoxification technology.

## 2. Zearalenone (ZEA) and Its Toxicity to Animals

### 2.1. Metabolic Structure of ZEA in Animals

There are two main ways that ZEA is transformed in the body. One involves the reduction to α-/β-zearalenol (α-/β-ZEL) under the catalytic action of 3α-/3β-hydroxysteroid dehydrogenase, and then a further reduction to α-/β-zearalanol (α-/β-ZAL) [23,24]. The other involves converting ZEA and its metabolites into glucuronic acid or sulfate metabolites under the action of uridine diphosphate glucuronyl transferase or sulfonyltransferase [25,26,27].

### 2.2. Toxic Damage in Animals Due to ZEA

#### 2.2.1. Reproductive Toxicity

ZEA and its metabolites can bind competitively to estrogen receptors (ERs) which subsequently activate estrogen response elements, resulting in the dimerization of the receptors and a variety of induced estrogenic effects [28,29]. In addition, the competitive binding products can also bind to the DNA template through the regulation of uterine target gene transcription and protein synthesis. This results in animal reproductive hormone disorders, thus affecting the animal’s reproductive development system [30,31,32]. Studies have shown that exposure to ZEA in the early pregnancy stage can affect the development of the placenta and embryo, and even lead to the deformity or death of the embryo [33].

#### 2.2.2. Cytotoxicity

ZEA can bind to the ERs in cytoplasm and result in lipid peroxidation (lipid peroxide can cause serious damage to cell membranes, lipoproteins, and other organelles and cell components containing lipid structures) which then produces a series of cytotoxic effects [34,35]. Early studies show that ZEA-induced apoptosis is related to mitochondrial apoptosis or the endoplasmic reticulum stress pathway, which is characterized by the mass production of reactive oxygen species and aggravation of lipid peroxidation [36,37]. In recent years, it has been confirmed that the apoptosis of kidney cells in mice due to the presence of ZEA in their diet are caused by the activation of the endoplasmic reticulum stress pathway [17].

#### 2.2.3. Immunotoxicity

ZEA can also bind to ERs on the surface of the cells of the immune system and thus regulate a variety of metabolic pathways of the immune response. It has been confirmed that ZEA not only activates immune response-related genes, but also interferes with the immune system of the spleen, changes the phenotypes of spleen lymphocytes, and even causes lymphocyte atrophy [38,39]. In addition, ZEA can induce immunosuppression by reducing immunoglobulins in serum and cytokines in lymphoid organs [40].

## 3. Non-Pathogenic Microorganisms with Detoxification EFFECTS toward Zearalenone

Biological detoxification is the most widely used detoxification method at present, which has become the main trend of mycotoxin detoxification research in recent years. At present, a variety of non-pathogenic microorganisms have been reported that can be used for the detoxification of ZEA, as shown in Table 1.

## 4. Biological Detoxification of ZEA

The biological detoxification method mainly involves the adsorption of the mycotoxin onto the walls of the microbial cells or degradation of the mycotoxin caused by microbial secretases. ZEA decontamination using non-pathogenic microorganisms is the main trend of research at present and promises to be a new way of achieving mycotoxin detoxification in practical situations in the future.

### 4.1. Adsorption of ZEA by the Cell Walls of Specific Strains

Special structures in the cell walls of certain probiotics allow them to adsorb the ZEA toxin. This reduces the exposure of the animal to ZEA which thus achieves the detoxification required. For example, cell walls contain carbohydrates (peptidoglycan, mannose, glucan), proteins, and lipids, which may present a variety of different adsorption centers (and so there may be various adsorption mechanisms involved, e.g., hydrogen bonding, ionic interactions, or hydrophobic interactions). The development of probiotics as mycotoxin-adsorbing agents and their potential use in production practice were thus prospected.

The results of this investigation show that yeasts are relatively stable mycotoxin-adsorbing agents, and that the main body responsible for this adsorption is the functional carbohydrates (glucomannan polymers) in their cell walls. In addition, adding 0.2% yeast cell wall extract to feed effectively prevented reproductive toxicity induced by 0.4 mg/L ZEA in piglets [55,56]. The ability of *Saccharomyces cerevisiae* to bind the mycotoxin to its cell walls has been evaluated. The cell diameter/cell wall thickness relation showed a correlation between the cell wall surface area and ZEA removal ability [53]. This proves that physical adsorption is the main mechanism responsible for the removal of the ZEA in this instance. Yiannikouris et al. investigated the correlation between the amount of β-D-glucan in the cell walls of *S. cerevisiae* and mycotoxin binding ability and found that the β-D-glucan plays a major role in ZEA adsorption [57]. Further experiments were performed to compare the ability of the yeast cell wall (YCW) extract and hydrated calcium aluminosilicate to adsorb ZEA. It was found that the YCW extract adsorbed ZEA more effectively in the gastrointestinal tracts of monogastric animals. Moreover, it was able to adsorb 40% of the total ZEA content in the intestines [58].

In recent years, *S. cerevisiae* has been continuously developed as a nutritional additive and has been added to feed in production practice to act as a detoxification agent inhibiting ZEA toxicity. Investigations have shown that the colonization of *S. cerevisiae* in the gastrointestinal tract not only improves the productivity and health of the animals, but also minimizes the bioavailability of ZEA in the tract [59,60]. Therefore, in terms of mycotoxin-adsorbing agents, the cell wall extracts and living cells of *S. cerevisiae* have become the focus of mycotoxin detoxification research. They are also proving to be a good tool for the sustainable development of modern animal husbandry.

The ability of some *Lactobacillus* strains to adsorb ZEA has also been demonstrated. For example, the ZEA (2.0 μg/mL) were incubated with either *L. rhamnosus* strain, a considerable proportion (38% to 46%) of ZEA toxin was recovered from the bacterial pellet [61]. Although used as a food additive, *L. rhamnosus* was able to effectively remove mycotoxins from the feed and promote the immune barrier of the host [62]. From using heat and acid treatment of the cell walls, it was further found that the thickness of the cell walls was positively correlated with the adsorption capacity towards ZEA [51]. Meanwhile, *L. plantarum* has also been confirmed to have great potential as a ZEA absorbent [49].

Some *Bacillus* spp. strains also have the ability to adsorb ZEA [42,44,45]. However, in terms of mycotoxin detoxification, the adsorption capacity of almost all of the *Bacillus* spp. strains is far less important than their degradation effects caused by secretase. Subsequently, most studies have paid more attention to researching and developing their use in degradation enzyme technology.

### 4.2. ZEA Biotransformation

The term biotransformation refers to the way in which microorganisms are able to change the molecular structure of ZEA in the process of metabolism. The possible pathways available for ZEA bioconversion relate mainly to the reduction of the ketonic carbonyl group, modification of the phenolic hydroxyl group, hydrolysis of the lactone ring, and cracking of the dihydroxybenzene ring.

#### 4.2.1. Occurrence of Secondary Metabolites of ZEA

**Figure 1:**
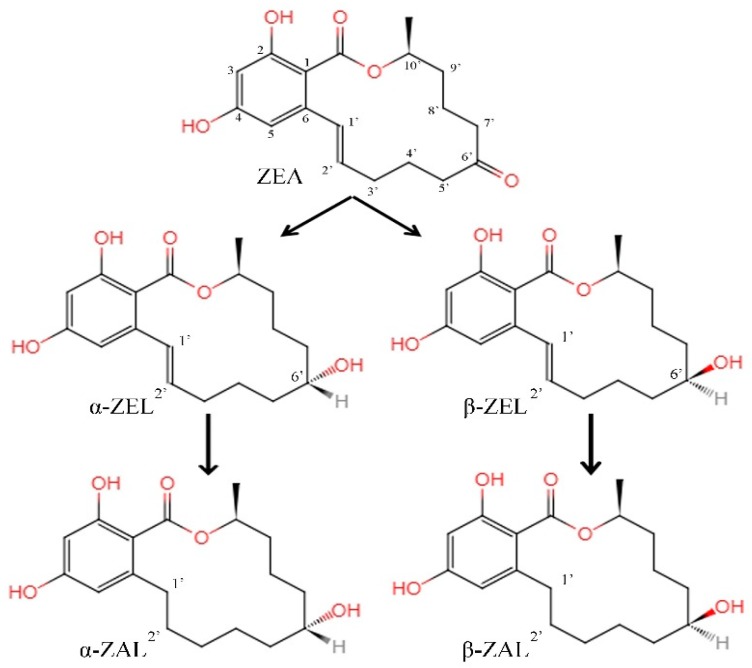
The formation pattern of secondary metabolites of zearalenone. ZEA: Zearalenone. α-/β-ZEL: α-/β-zearalenol. α-/β-ZEL: α-/β-zearalanol.

The C_6’_-ketonic carbonyl group in ZEA is readily reduced. Thus, it is very easy to add hydrogen atoms to generate zearalenol (α-/β-ZEL). Further reduction leads to the disappearance of the C_1’_=C_2’_ double bond, generating the corresponding zearalanol (α-/β-ZAL), as shown in Figure 1.

It has been reported that, *Fusarium roseum* ‘*gibbosum*’ can convert ZEA into α/β-ZEL [63]. A mixed culture of *Candida tropicalis*, *Zygosaccharomyces rouxii*, and 7 *Saccharomyces* strains have also been reported to accomplish the same conversion [64]. Furthermore, ZEA can be reduced to α-ZEL (and β-ZEL to a lesser extent) under the action of rumen microorganisms [65]. As the toxicity of α-ZEL toward estrogen is higher than ZEA, its formation can be more damaging to the reproductive system of the livestock [23].

#### 4.2.2. Modification of the Phenolic Hydroxyl Groups in ZEA

The phenolic hydroxyl groups (C_2_/C_4_-OH) in ZEA can also be readily oxidized by a variety of oxidants. It has been reported that *Rhizopus* spp. catalyzed the glycosylation of ZEA at the C_4_-OH group to form a new metabolic structure called zearalenone-4-beta-D-glucopyranoside [66]. *Fusarium* spp. (*F. roseum*, *F. equiseti*, and *F. sambucinum*) and *Rhizopus arrhizus* catalyze the sulfation of ZEA at the C_4_-OH group to form sulfate metabolites of ZEA, i.e. zearalenone-4-sulfate [67,68]. When ZEA was biotransformed using *Aspergillus* and *Rhizopus* species, however, it was found that ZEA-4-sulfate was only formed when *A. oryzae* was used and ZEA-4-glucosides and ZEA-2-glucosides were formed by *R. oryzae* and *R. oligosporus*, respectively [69]. It is worth noting that α-ZEL-sulfate also occurred in the modified ZEA products during transformation with *R. oryzae* [70].

In studies on ZEA modification pathways, *Arabidopsis* UDP-glucosyltransferases has been widely used to produce zearalenone-4-O-glucosides [71,72]. Synthesizing ZEA-glucosides using recombinant barley glucosyltransferase (HvUGT14077) showed that the ZEA was converted effectively to ZEA-4-glucosides and ZEA-2-glucosides. However, ZEA-2,4-di-glucosides, α-/β-ZEL-2-glucosides, α-/β-ZEL-4-glucosides, and α-/β-ZEL-2,4-di-glucosides were also synthesized at the same time [73]. However, these modifications of ZEA cannot be considered to be an effective way of detoxifying ZEA in vivo. This is because studies have shown that ZEA-4-sulfate, ZEA-4-O-β-glucosides, and ZEA-2-O-β-glucosides can be completely hydrolyzed in the gastrointestinal tract of pigs which can re-release ZEA and other unknown metabolites [24,25]. Table 2 displays the molecular structures of ZEA and its phenol hydroxy (C_2_/C_4_-OH) derivatized products.

#### 4.2.3. Formation of Non-Toxic Degradation Products from ZEA

ZEA and its derivatives exhibit estrogenic activity because their chemical structures are similar to natural estrogen. From the point of view of molecular structure, therefore, the existing lactone structure of ZEA can be changed so that it no longer has estrogenic effects. This can be achieved by hydrolyzing the lactone ring, breaking the C_6’_-ketonic carbonyl group, or cracking the dihydroxybenzene ring. These changes are also often referred to as degradation pathways of ZEA.

**Figure 2:**
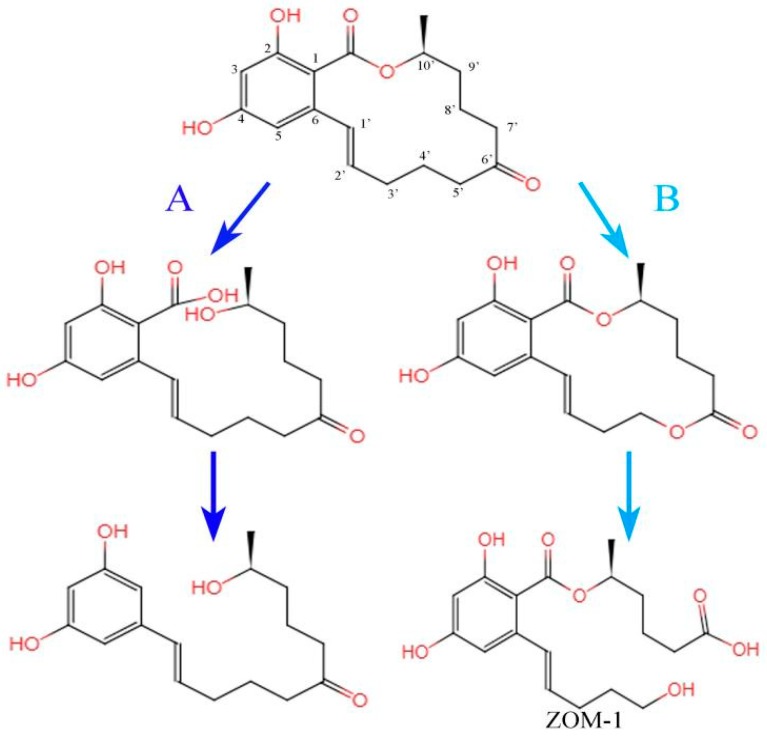
Biotransformation diagrams for zearalenone. (**A**) The hydrolysis of lactone ring. Final product: 1-(3,5-dihydroxyphenyl)-6’-hydroxy-l’-undecen-l0’-one. (**B**) The cracking of ketone carbonyl group. ZOM-1: (5S)-5-({2,4-dihydroxy-6-[(1E)-5-hydroxypent-1-en-1-yl]benzoyl}oxy)hexanoic acid.

The ester group in the lactone ring of ZEA is easily hydrolyzed by esterase or acid-base hydrolysis and then ring-opening. For example, it has been reported that *Gliocladium roseum* is able to hydrolyze the lactone bond of ZEA to produce a kind of 1-(3,5-dihydroxyphenyl)-10′-hydroxy-1-undecen-6-one which has no estrogenic toxicity [74]. Takahashi-Ando found that the protein encoded by the zearalenone lactonohydrolase gene ZHD101 can degrade ZEA [75]. Further studies indicated that the lactonohydrolase breaks the lactone bond in the ZEA structure. The ring structure thus opened up into a straight chain structure which was subsequently decarboxylated (Figure 2, reaction pathway A). The decarboxylated product could not bind to the ERs, so the toxicity of ZEA was reduced or even completely eliminated [76,77]. It has also been proved that ZHD101 is the only α/β-hydrolase which could be used to detoxify ZEA and its derivatives [78,79].

Similar ZEA degradation pathways may also exist when *Bacillus* spp. are used. For example, ZEA can be degraded using culture extracts from *B. subtilis* 168 and *B. natto* CICC 24640. The rate of ZEA degradation using *B. subtilis* 168 and *B. natto* CICC 24640 culture extract after 24 h of aerobic incubation at 30 °C was found to be 81% and 100%, respectively [42]. The process was accompanied by the release of CO_2_, thus indicating that decarboxylation had occurred. An analysis of the ZEA degradation products produced by *B. pumilus* showed that 1-(3,5-dihydroxyphenyl)-6′-hydroxy-l′-undecen-l0′-one had been produced [80]. This led to the speculation that the reaction involved the catalytic hydrolysis of the ZEA lactone ring by lipase.

With the development of transgenic technology, studies have found that when the isolated lactonohydrolase gene ZHD101 was recombined with yeast cells, the recombinant gene was successfully expressed in the yeast and the expressed enzyme degraded ZEA very well [81]. ZHD101 has also been introduced into *L. reuteri* Pg4. The result, *L. reuteri* pNZ-zhd101, was able to successfully express the ZHD101 gene and thus acquired the ability to degrade ZEA [50]. In fact, this is the first report of the successful expression of a ZEA-degrading enzyme by an intestinal probiotic. The application of transgenic technology to ZEA degradation has great potential in the field of ZEA detoxification. It also promises to have further production and practical applications.

In biochemistry, carbonyl groups can be readily oxidized to form lipids under the action of Baeyer-Villiger monooxygenase. This involves the conversion of ketones to esters or cyclic ketones to lactones via the introduction of an oxygen atom into the ortho-position of the carbonyl group [82]. Similar reactions have been shown to occur in the biodegradation of ZEA. For example, studies showed that *Apiotrichum mycotoxinivorans* opens the C_6’_-ketone carbonyl group of ZEA to form carboxy and hydroxyl groups. The reaction starts with the formation of a new lactone by the addition of an oxygen atom to the C_6’_-ketone carbonyl group. This subsequently undergoes hydrolysis to produce ZOM-1 under the action of hydrolase (Figure 2, reaction pathway B). It has been confirmed that ZOM-1 has no estrogenic effect in vivo and does not interact with ER protein in vitro. Based on this, the identification of the key genes or degradation enzymes involved in the detoxification of ZEA by *Apiotrichum mycotoxinivorans* can also provide new insights into the detoxification pathways available for ZEA [83].

ZEA is a mycotoxin with a dihydroxybenzoic acid lactone structure. Therefore, the cracking of the dihydroxybenzene ring can also be expected to constitute an effective method of detoxification. It has been reported that the fermentation of *Aspergillus niger* strain FS10 significantly decreases the ZEA content (29 μg/mL) in corn pulp. A subsequent analysis based on HPLC-MS and UV-vis spectroscopy showed that the ZEA was transformed into two metabolites: ZEA-A (*m*/*z* = 414) and ZEA-B (*m*/*z* = 325). ZEA-B does not absorb UV, indicating that the ring structure in ZEA may have been destroyed in this metabolite [84].

Coincidentally, other studies have also shown that *Acinetobacter* spp. SM04 degraded ZEA into ZEA-1 (*m/z* = 489) and ZEA-2 (*m/z* = 405), both of which have UV absorption spectra that are different to that of ZEA. The 3-(4,5-dimethylthiazol-2-yl)-2,5-diphenyltetrazolium bromide assays also showed that the degradation products had no estrogenic activity with respect to MCF-7 cells. Hence, it was inferred that the benzene ring in ZEA had been oxidized and broken to form compounds containing carboxy groups [85]. The peroxiredoxin (Prx) isolated from *Acinetobacter* spp. SM04 has also been successfully expressed in *Escherichia coli* and *Pichia pastoris*, and the Prx enzyme was thus found to degrade ZEA into two products [86,87]. Thus, further developing such dihydroxybenzene ring opening reaction pathways can also be expected to become a new direction to enhance the biological detoxification of ZEA.

To sum up, the important future breakthroughs in the field of ZEA biodegradation are most likely to result from: (i) Finding and screening better ZEA-degrading strains; (ii) studying the characteristics of the ZEA-degrading enzymes produced by these ZEA-degrading strains; and (iii) cloning and expressing these degrading enzyme genes.

#### 4.2.4. Unknown ZEA Detoxification Pathways

A large number of microorganisms capable of degrading ZEA have been reported to date. However, in the case of ZEA detoxification using non-pathogenic microorganisms, the detoxification mechanism has only been identified for a few ZEA-degrading bacteria, that is, most ZEA-degrading bacteria act via detoxification mechanisms that have not been clarified.

The identification of a non-pathogenic *Rhodococcus pyridinivorans* K408 strain proved to be a new type of ZEA-degrading strain. In this particular case, after Luria-Bertani broth contaminated with ZEA (500 mg/L) was treated with *R. pyridinivorans* K408 strain for five days, the degradation efficiency with respect to ZEA was found to be 87.2%. Although the degradation products created during the ZEA biotransformation process are not clear, the strain does not produce any metabolites with estrogenic effects [88]. A new strain of *S. cerevisiae* has also been found that is able to degrade rather than adsorb ZEA. In this case, *S. cerevisiae* was cultured in nutrient yeast dextrose broth containing ZEA for 48 h, after which, the ZEA (5 mg/mL) was found to be completely degraded by the *S. cerevisiae* [89]. When *Bacillus* strains are used for the biological detoxification of ZEA, the active degradation enzymes secreted by most strains clearly play a leading role in the degradation process. However, the active degradation enzymes and degradation mechanisms involved are not clear. For example, *Bacillus subtilis* and *B. amyloliquefaciens* had been found to effectively degrade ZEA and its derivatives under optimum reaction conditions. Furthermore, it has been preliminarily determined that the extracellular enzymes secreted by bacteria played a dominant role in the detoxification of ZEA [41,42,45].

*B. velezensis* A2 was able to completely degrade ZEA (7.45 μg/mL) after three days of incubation in Luria-Bertani medium. Although the degradation mechanism is unclear, it was found that using *B. velezensis* A2 as a food additive was effectively able to purify feed contaminated with ZEA and protect mice from the toxic effects of ZEA [37,46].

In summary, further studies focusing on the structure, toxicity, and degradation mechanisms of ZEA metabolites can be expected to further enhance the development of new and improved microbial ZEA-detoxification strategies.

## 5. Development Trends in Probiotic ZEA Decontamination

Probiotics have broad application prospects as mycotoxin detoxification agents. Indeed, their application in the field of mycotoxin decontamination is changing with each passing day. To date, the expression of ZEA-degrading enzyme genes in probiotic host cells has been the main focus of their use in ZEA toxin decontamination (ultimately aimed at ensuring the safety of food intended for consumption by animals and humans). However, when food/feed is assessed to determine its safety, it is not uncommon to find more than one type of mycotoxin in a given sample. Thus, it is becoming progressively more important to aim for the simultaneous degradation of a variety of mycotoxins [90,91,92].

It was reported in a study involving the adsorption of ZEA by *Lactococcus lactis* and a *Bifidobacterium* sp., that the absorption process was not homogeneous, that is, the ZEA was adsorbed in two stages: An initial adsorption stage that was rapid; and a subsequent adsorption stage that was much slower [93]. It has also been shown that a microbial community composed of a variety of mycotoxin-degrading bacteria was capable of simultaneously degrading aflatoxin B1 and ZEA [94]. Moreover, there appeared to be a synergistic effect between the degrading strains that promoted the efficient detoxification of the mycotoxins.

In recent years, the combined use of compound probiotics (*Bacillus* spp., *Lactobacillus* spp., and yeast) and mycotoxin-degrading enzymes has also proved to have a simultaneous detoxification effect on AFB1 and ZEA [95,96]. It has also been confirmed that compound probiotics not only increased the rate of degradation of ZEA, but also made the intestinal epithelial barrier more resistant to being damaged by mycotoxins and other pathogenic microorganisms [97]. In short, the combined use of compound probiotics and mycotoxin-degrading enzymes constitutes yet another new strategy for mycotoxin decontamination.

## 6. Epilogue

As the biological detoxification of zearalenone is studied in greater depth, more and more novel probiotic strains (*Bacillus* spp., *Lactobacillus* spp., and yeast) and degradation enzymes (lactone hydrolase, peroxidase) are likely to be discovered. Subsequently, the mechanism(s) by which these probiotics detoxify ZEA will gradually become well known and their use as feed/food additives can be mastered and perfected.

Currently, however, biological detoxification technology is at a stage where it is still imperfect but it is continuing to mature. Thus, the production of enzyme and microbial preparations (and other biological additives) is rapidly moving towards the stage where it can be industrialized.

## Figures and Tables

**Table 1:** Non-pathogenic microorganisms that can be used for the detoxification of Zearalenone (ZEA).

Microorganism		Authors/Refs.
*Bacillus* spp.	*B. subtilis*	Cho [41], Tinyiro [42]
	*B. licheniformis*	Fu [43], Hsu [44]
	*B. amyloliquefaciens*	Lee [45]
	*B. velezensis*	Wang [17,46]
	*B. cereus*	Wang [47]
*Lactobacillus* spp.	*L. rhamnosus*	El-Nezami [48]
	*L. plantarum*	Vega [49]
	*L. reuteri*	Yang [50]
	*L. mucosae*	Long [51]
	*L. paracasei*	Abbès [52]
*Saccharomyces* spp.	*S. cerevisiae*	Armando [53], Krifaton [54]

**Table 2:** The structure of ZEA and its phenol hydroxy (C_2_/C_4_-OH) derivatives.

Action Site Diagram			
Molecule	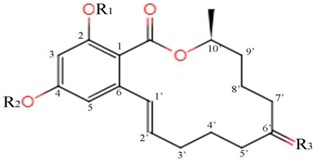		
	R1	R2	R3
ZEA	H	H	O
ZEA-2-O-β-glucosides	glucose	H	O
ZEA-4-O-β-glucosides	H	glucose	O
ZEA-2,4-di-glucosides	glucose	glucose	O
ZEA-4-sulfate	H	sulfate	O
α-ZEL-2-glucosides	glucose	H	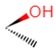
β-ZEL-2-glucosides	glucose	H	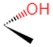
α-ZEL-2,4-di-glucosides	glucose	glucose	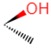
β-ZEL-2,4-di-glucosides	glucose	glucose	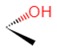
